# A Comprehensive Review on Bio-Based Polybenzoxazines Emphasizing Their Antimicrobial Property

**DOI:** 10.3390/microorganisms13010164

**Published:** 2025-01-14

**Authors:** Shakila Parveen Asrafali, Thirukumaran Periyasamy, Jaewoong Lee

**Affiliations:** Department of Fiber System Engineering, Yeungnam University, Gyeongsan 38541, Republic of Korea; shakilaasraf@gmail.com (S.P.A.); thirukumaran@ynu.ac.kr (T.P.)

**Keywords:** polybenzoxazine, synthesis, antimicrobial properties, applications

## Abstract

Polybenzoxazines (PBzs), a class of high-performance thermosetting polymers, have gained significant attention for their exceptional thermal stability, mechanical properties, and chemical resistance, making them ideal for aerospace, electronics, and biomedical applications. Recent advancements emphasize their antimicrobial potential, attributed to unique structural properties and the ability to incorporate bio-active functional groups. This review highlights the synthesis, antimicrobial mechanisms, and applications of PBzs and their bio-based derivatives, focusing on sustainable materials science. PBzs demonstrate antimicrobial efficacy through mechanisms such as hydrophobic surface interactions and reactive functional group formation, preventing microbial adhesion and biofilm development. The incorporation of functional groups like amines, quaternary ammonium salts, and phenolic moieties disrupts microbial processes, enhancing antimicrobial action. Modifications with metal nanoparticles, organic agents, or natural bio-actives further augment these properties. Notable bio-based benzoxazines include derivatives synthesized from renewable resources like curcumin, vanillin, and eugenol, which exhibit substantial antimicrobial activity and environmental friendliness. Hybrid PBzs, combining natural polymers like chitosan or cellulose, have shown improved antimicrobial properties and mechanical performance. For instance, chitosan-PBz composites significantly inhibit microbial growth, while cellulose blends enhance film-forming capabilities and thermal stability. PBz nanocomposites, incorporating materials like silver nanoparticles, present advanced applications in biomedical and marine industries. Examples include zirconia-reinforced composites for dental restoration and urushiol-based PBzs for eco-friendly antifouling solutions. The ability to customize PBz properties through molecular design, combined with their inherent advantages such as flame retardancy, low water absorption, and excellent mechanical strength, positions them as versatile materials for diverse industrial and medical applications. This comprehensive review underscores the transformative potential of PBzs in addressing global challenges in antimicrobial material science, offering sustainable and multifunctional solutions for advanced applications.

## 1. Introduction

**Scheme 1:**
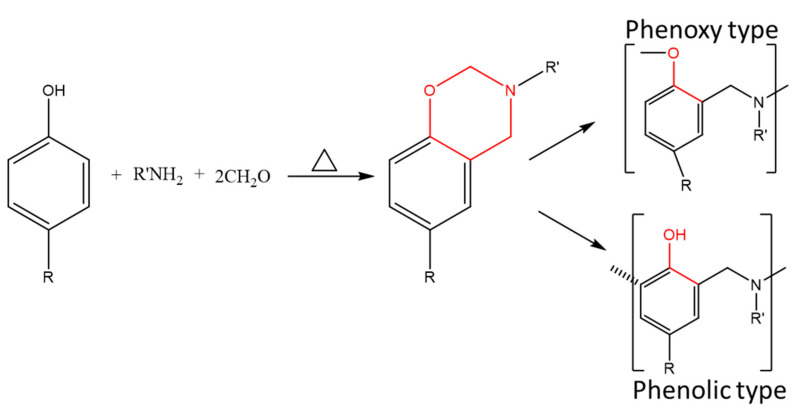
General synthesis of benzoxazine monomer and their polymerization.

Polybenzoxazines (PBzs) are a class of high-performance thermosetting polymers known for their exceptional thermal stability, mechanical properties, and diverse applications. These polymers exhibit low water absorption, excellent dimensional stability, and high resistance to chemicals, making them ideal for aerospace, electronics, and sustainable materials development. Recent research has highlighted innovative formulations and applications of polybenzoxazines, focusing on sustainability; nanocomposites; thermal and mechanical performance; shape memory; and smart materials. Ring-opening polymerization (ROP) of polybenzoxazines involves the transformation of benzoxazine monomers into thermosetting polymers through a thermal curing process (Scheme 1).

This method provides polybenzoxazines with unique structural features, such as high thermal stability, flame retardancy, and excellent mechanical properties [1,2,3,4,5,6]. The polymerization of 3,4-dihydro-1,3-benzoxazines can produce two distinct polymer structures, determined by the nucleophilic atom attacking the electrophilic methylene group (NCH_2_O). When the phenolic oxygen performs the attack, the process initially forms N, O-acetal linkages (phenoxy-type structures), which can subsequently rearrange into Mannich-type structures with free phenolic groups (phenol-type structures). Alternatively, an attack by a phenolic carbon at the ortho or para positions of the benzoxazine directly generates phenolic-type structures. The intra- and intermolecular hydrogen bonding of phenolic units with other functional groups—such as amine, ether, alcohol, or aromatic groups—plays a crucial role in determining the performance characteristics of polybenzoxazines [7,8,9].

**Figure 1:**
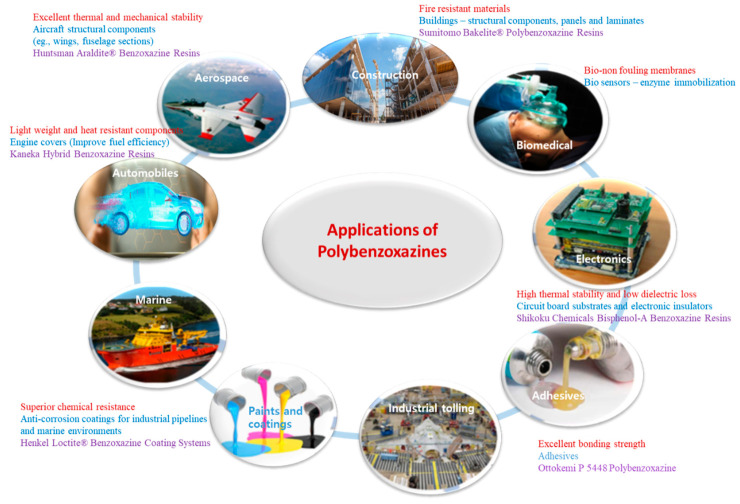
Applications of polybenzoxazines in various fields.

PBzs possess several advantages that make them highly desirable for various industrial and scientific applications, which include: (i) Superior thermal properties—polybenzoxazines exhibit exceptional thermal resistance, with decomposition temperatures often exceeding 300 °C and a low coefficient of thermal expansion (CTE), making them suitable for electronic and aerospace applications. (ii) High mechanical strength—these materials possess excellent rigidity, toughness, and impact strength, making them ideal candidates for structural applications and composite reinforcements. (iii) Enhanced chemical resistance—polybenzoxazines are hydrophobic in nature, resulting in low water absorption and moisture resistance. They also resist a wide range of solvents, acids, and bases, making them suitable for harsh chemical environments. (iv) Enhanced electrical properties—they have low dielectric constants, making them ideal for electronic components and printed circuit boards. They also provide excellent electrical insulation, which is critical in high-voltage applications. (v) Intrinsic flame retardancy—their polymer backbone is inherently resistant to combustion, making them suitable for high-performance fire-resistant applications. (vi) Tailorable properties—utilizing molecular design flexibility benzoxazine monomers can be synthesized with specific functional groups, enabling the customization of mechanical, thermal, and chemical properties. Moreover, they are compatible with other fillers, fibers, and nanoparticles, enabling their property enhancement. (vii) Self-catalytic polymerization—polybenzoxazines undergo ring-opening polymerization without the need for external catalysts, simplifying processing and reducing costs [10,11,12,13,14,15,16,17]. Due to their enormous properties, PBzs find applications in several fields including aerospace and automotive industries, electronics, coatings and adhesives, fire-resistant materials, and biomedical applications (Figure 1).

PBzs have emerged as promising materials for antimicrobial applications due to their unique chemical structure and ability to inhibit microbial growth. The mechanism of antimicrobial action occurs through hydrophobic surfaces and by creating reactive functional groups. The hydrophobicity of polybenzoxazine-based coatings prevents microbial adhesion and biofilm formation, which is crucial for long-term antimicrobial efficacy. Polybenzoxazines synthesized with functional groups such as amines, quaternary ammonium salts, or phenolic moieties interfere with microbial metabolic processes by disrupting enzyme activity or cell wall integrity, thereby inhibiting microbial growth. PBzs can be modified with metal.

**Figure 2:**
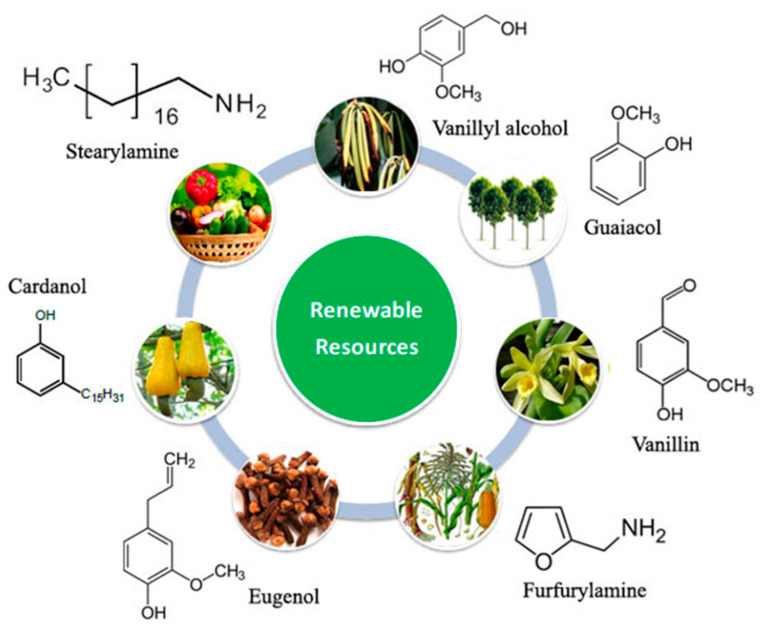
Renewable resources used for benzoxazine synthesis.

Nanoparticles (e.g., silver, copper), organic antimicrobial agents, or bio-active compounds enhance their effectiveness against bacteria, fungi, and viruses. Recent research focusing on synthesis strategies creating PBzs with antimicrobial properties include: (i) Synthesizing bio-based benzoxazines: benzoxazines synthesized from renewable resources, such as cardanol, eugenol, vanillin, curcumin, arbutin, tyrosine, magnolol, urushiol, furfural, and furfurylamine, exhibit antimicrobial and environmentally friendly properties (Figure 2); (ii) Nanoengineered PBz: embedding nanostructures like silver or zinc oxide nanoparticles enhances antimicrobial and mechanical properties; and (iii) Fabrication of hybrid PBz: combining PBzs with other polymers or copolymers (such as chitosan, cellulose, PEG—polyethylene glycol or its copolymers) possessing antibacterial and antifungal properties yields multi-functional materials suitable for advanced biomedical and industrial applications [18,19,20,21,22,23,24,25,26]. This review details the antimicrobial properties of PBzs through the above-mentioned strategies, covering a wide range of aspects, from their fundamental properties and mechanisms of antimicrobial action to their applications and future potential.

## 2. Antimicrobial Properties of Bio-Based PBzs

Bio-based benzoxazines have demonstrated potential as antimicrobial agents, leveraging their structural modifications and incorporation of bio-active compounds. These materials are particularly promising in applications requiring sterility, such as coatings, medical devices, and water purification systems. The integration of naturally occurring bio-active compounds such as curcumin, vanillin, eugenol, apigenin, furfurylamine, etc., and the design of benzoxazines further augment their antimicrobial efficacy.

### 2.1. Curcumin-Based Benzoxazines

**Figure 3:**
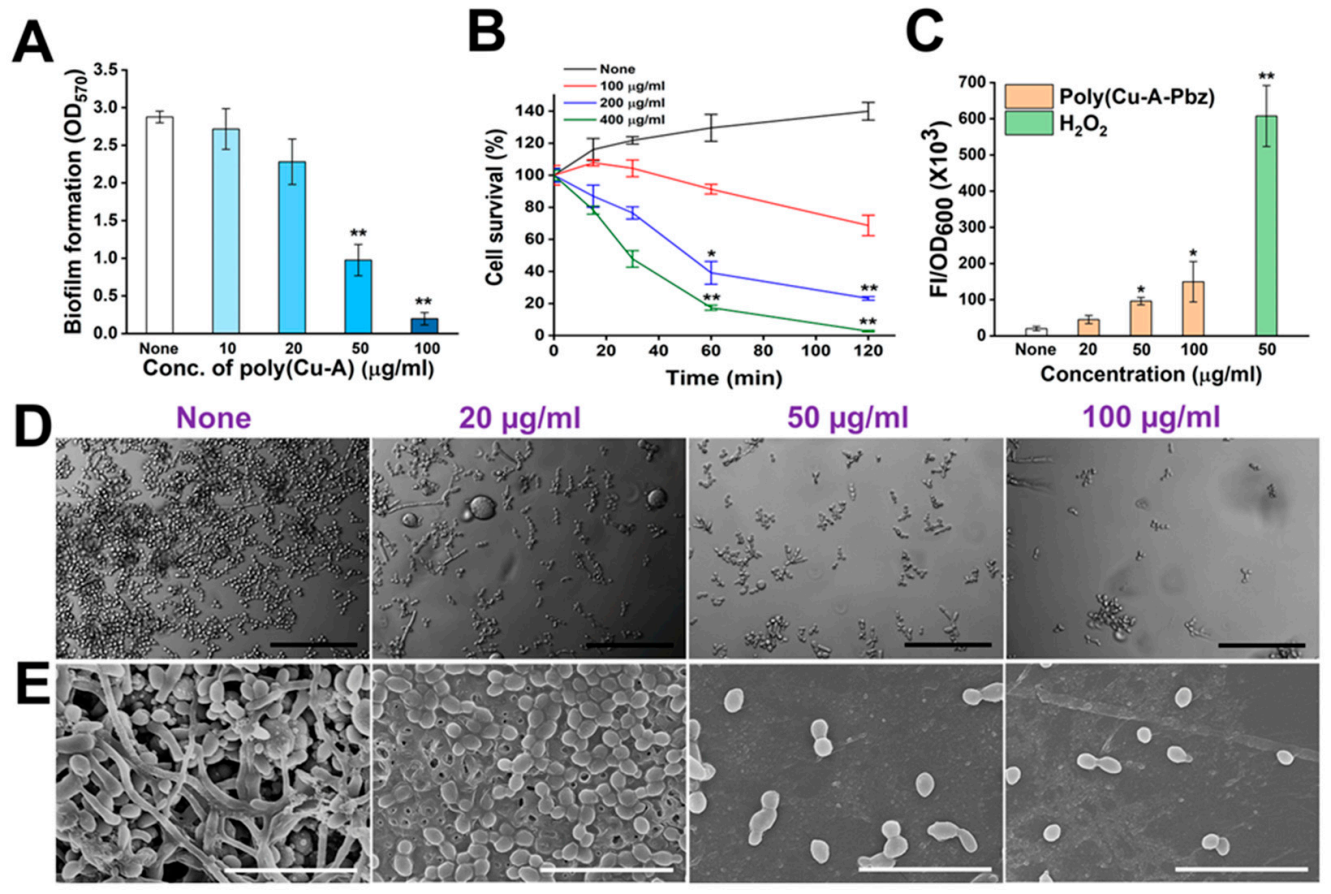
Evaluation of antifungal and antibiofilm potency of poly(Cu-A) compared to *C. albicans* DAY185: (**A**) biofilm formation, (**B**) rapid killing, (**C**) Poly(Cu-A)-induced ROS production, (**D**,**E**) SEM analysis of *C. albicans* biofilm against control and Poly(Cu-A) (scale bars: black = 100 μm, white = 20 μm) [35].

Curcumin, also known as 1,7-bis-(4-hydroxy-3-methoxyphenyl)-1,6-heptadiene-3,5-dione, is a yellow polyphenolic compound that can be extracted from rhizomes of turmeric (*Curcuma longa*). It is widely utilized in the medicinal field for its antioxidant, antibacterial, antiviral, and anti-inflammatory properties [27,28]. Furthermore, the presence of –OH groups in its structure makes curcumin suitable for synthesizing various monomers or blending with polymers that require hydroxyl functional groups [29,30,31,32,33,34]. Thirukumaran et al. [35] synthesized a novel curcumin-based PBzs [poly(Cu-A)] to adopt the properties of curcumin into the PBz network. A biofilm assay was conducted to evaluate the antibiofilm efficacy of Poly(Cu-A) against *C. albicans*. Poly(Cu-A) treatments at concentrations of 20, 50, and 100 µg/mL demonstrated a dose-dependent inhibition of biofilm formation. At 100 µg/mL, biofilm inhibition exceeded 92%, with minimal impact on cell growth. Optical microscopy and SEM images revealed a significant reduction in cell aggregation caused by Poly(Cu-A) (Figure 3). The environmental toxicity of Poly(Cu-A) was assessed using *C. elegans* and seed germination assays. More than 84% of the worms survived across various concentrations of Poly(Cu-A), indicating no toxic effects of the polymer composite. Additionally, Poly(Cu-A) did not induce phenotypic changes in seed germination over three days. However, at 200 µg/mL, the germination rate of *R. raphanistrum* was delayed after seven days. They also fabricated an entirely bio-based PBzs from curcumin and furfurylamine Poly(C-fu) and studied their antibacterial property [36]. The antibacterial property of Poly(C-fu) film was tested against *E. coli* and *S. aureus* using the disk diffusion method. The bacterial growth inhibition zone for Poly(C-fu) was found to be 11 ± 1.1 for *S. aureus* and 8 ± 2.3 for *E. coli*.

**Scheme 2:**
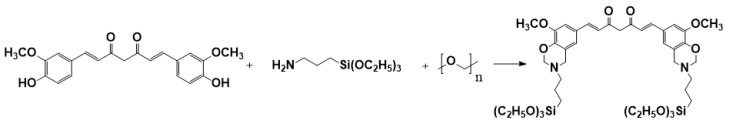
Synthesis reaction of the curcumin-based benzoxazine monomer (CB) [37].

Deng et al. [37] synthesized ecofriendly green coating by synthesizing bio-based high-performance benzoxazine (PCB) using curcumin, 3-aminopropyltriethoxysilane (APTES) and paraformaldehyde as raw materials (Scheme 2).

The synthesized biobenzoxazine imparts bio-activity from curcumin and improved cross-link density from two phenolic hydroxyl groups of curcumin and Si-O-Si framework from APTES. The antifouling performance of the coating was evaluated based on its ability to resist protein adsorption. A significant reduction in the adhered BSA-FITC (bovine serum albumin labeled by the fluorescein isothiocyanate) on the coating surface was observed, with the fluorescence intensity of the PCB coating decreasing to 51.17% compared to bare glass (100%). The antibacterial activity of the PCB coating was tested against *E. coli* and *S. aureus*. While a large number of aggregated bacteria adhered to the pristine glass, only a small amount was observed on the PCB coating, demonstrating its enhanced antibacterial properties. The antifouling behavior was further assessed using microalgae species such as *N. closterium* and *Dicrateria zhanjiangensis* as model fouling organisms. The coverage of these microalgae on the PCB coating was significantly reduced, showing antifouling efficiencies of 40.81% and 38.73%, respectively, compared to pristine glass.

### 2.2. Vanillin and Furfurylamine-Based Benzoxazine

**Scheme 3:**
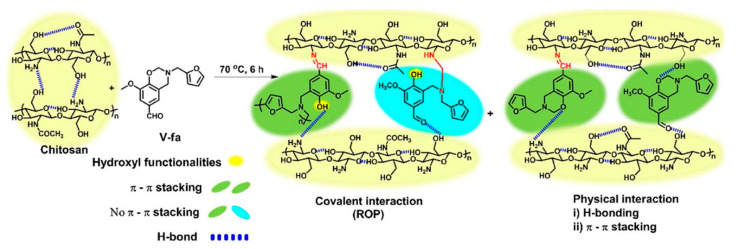
Probable different types of interactions of V-fa with chitosan [38].

Yadav et al. synthesized a fully bio-based benzoxazine monomer, V-fa, derived from vanillin and furfurylamine [38] (Scheme 3). Vanillin is sourced from vanilla pods and the naturally abundant biopolymer lignin, while furfurylamine is obtained through the chemical transformation of furfural-rich agricultural byproducts such as corncobs, bagasse, and wheat bran. The antibacterial activity of V-fa was tested against *S. aureus* UAMS-1, a common and highly virulent bacterial strain. The zone of growth inhibition around the film circle was measured in millimeters, with the diameter correlating to the bacterial susceptibility and the diffusion rate of the antibacterial species through the agar medium. Oligomerized V-fa exhibited slightly enhanced antibacterial activity compared to the V-fa monomer, attributed to the formation of a ring-opened oxazine structure, which generates phenolic and amine functionalities known for their antibacterial properties.

### 2.3. Arbutin-Based Benzoxazine

**Scheme 4:**
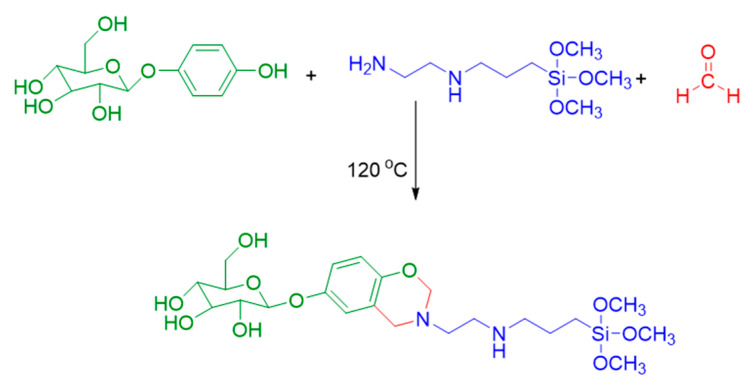
Synthesis of arbutin-based benzoxazine monomer (AB) [39].

Arbutin-based polybenzoxazine (PAB) was synthesized using arbutin, [3-(2-aminoethylamino) propyl] trimethoxysilane (AEAPTMS), and paraformaldehyde [39] (Scheme 4). Arbutin, also known as the β-d-glucoside of hydroquinone, is a natural compound with widespread availability from plant families such as *Ericaceae* (bearberry) and *Saxifragaceae* (herbaceous plants). Notably, arbutin requires no additional specific conditioning for use and is widely recognized for its ability to inhibit the melanin biosynthetic pathway, making it a popular skin-lightening agent in cosmetics. The antimicrobial activity of PAB was evaluated against *C. albicans*. At a concentration of 100 μg/mL, PAB demonstrated a significant reduction in biofilm mass, achieving a 72.7% decrease as measured by the crystal violet assay.

### 2.4. Apigenin-Based Benzoxazines

**Figure 4:**
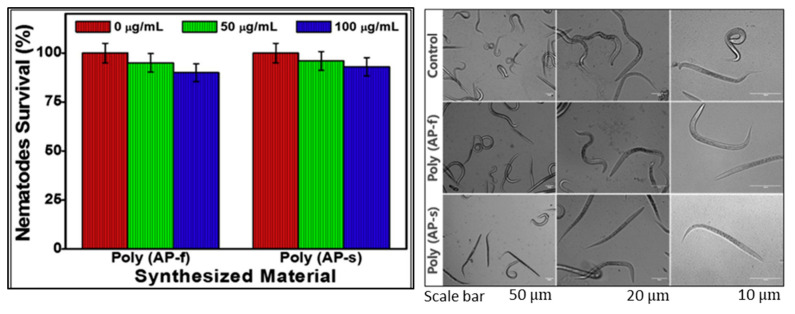
Nematode survival of [poly(AP-f) and Poly(AP-s)] with 0 (control) 50 and 100 μg/mL concentrations [40].

Apigenin was reacted with furfurylamine/stearylamine and formaldehyde to synthesize multifunctional bio-based benzoxazine monomers (AP-f and AP-s) [40]. Apigenin, a natural bioflavonoid, is abundant in vegetables such as parsley, fruits like grapes and apples, and beverages such as tea and red wine. Furfurylamine, a bio-based amine derived from furfural, is an agricultural byproduct obtained from corncobs and wheat bran. Stearylamine, sourced from vegetable oil, is part of the fatty amine family characterized by long hydrocarbon chains. Poly(AP-f) exhibited significant antibiofilm activity, reducing biofilm formation by *S. aureus* by more than 60% at 50 μg/mL and over 80% at 100 μg/mL. In contrast, Poly(AP-s) showed no notable inhibition of biofilm formation at either concentration against *S. aureus*. Toxicity assays performed on *C. elegans* revealed no adverse effects on nematode growth even after seven days of exposure, suggesting non-toxicity at both concentrations. Microscopic observations confirmed that the worms were alive, with survival rates exceeding 85% and 90% for Poly(AP-f) and Poly(AP-s), respectively, at a concentration of 100 μg/mL (Figure 4).

### 2.5. Eugenol Based Benzoxazine

A sustainable and eco-friendly method was employed to synthesize bio-based polybenzoxazines using eugenol, chitosan, and formaldehyde (E-ch) [41]. Eugenol, a naturally occurring phenolic compound, is abundant in clove oil and other plant sources. The antimicrobial properties of poly(E-ch) were assessed against *S. aureus* and *C. albicans*. The poly(E-ch) film demonstrated significant activity against both microorganisms at concentrations of 50 and 100 μg/mL. Furthermore, toxicity evaluations using *C. elegans* showed no adverse effects on nematode growth after seven days of exposure, indicating that the synthesized film is non-toxic.

### 2.6. Tyrosine and Furfural Based Benzoxazines

**Figure 5:**
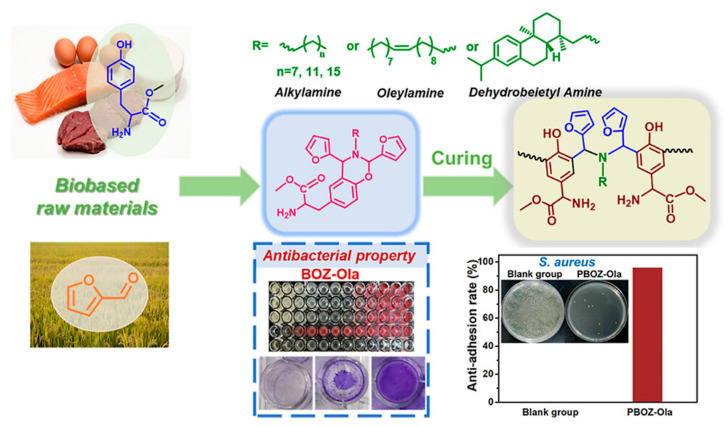
Bio-based benzoxazines from L-tyrosine methyl ester and furfural [42].

**Figure 6:**
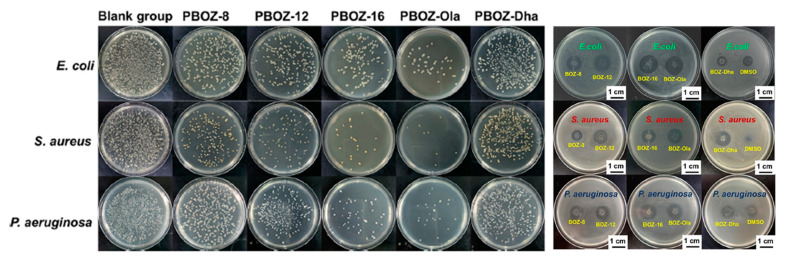
Antiadhesion property of PBOZs and inhibition zones of BOZs [42].

Tyrosine and its derivatives are aromatic amino acids found in various proteins, and serve as phenolic sources that can replace petroleum-based counterparts to create sustainable materials for functional applications. Yuan et al. [42] synthesized a series of bio-based benzoxazine monomers (TF-BOZs) using tyrosine methyl ester, furfural, and five amine sources (octylamine, dodecylamine, 1-hexadecylamine, oleylamine, and dehydroabietylamine) through the Mannich condensation reaction (Figure 5). The antibacterial efficacy of TF-BOZs was assessed against *S. aureus*, *E. coli*, and *P. aeruginosa* using double dilution and plate counting methods to determine minimum inhibitory concentrations (MICs) and minimum bactericidal concentrations (MBCs). The results revealed that antibacterial activity improved significantly with longer alkyl chain lengths. This enhancement is attributed to the electrostatic attraction of BOZs to negatively charged bacterial surfaces and the ability of longer alkyl chains, with greater lipophilicity, to penetrate and disrupt bacterial cell walls via hydrophobic interactions, leading to intracellular substance leakage. Among the monomers, BOZ-Ola demonstrated the best antibacterial performance, with MIC and MBC values of 5 and 110 μg/mL against S. aureus, 17 and 360 μg/mL against *E. coli*, and 53 and 780 μg/mL against P. aeruginosa. Furthermore, PBOZ-Ola exhibited excellent anti-adhesion properties, achieving a 90% reduction in bacterial adhesion against *E. coli*, *S. aureus,* and *P. aeruginosa* (Figure 6).

## 3. Antimicrobial Properties of Hybrid PBzs

Combining PBzs with other polymers has gained significant attention in material science, driven by the need for sustainable, biodegradable, and high-performance materials. Both chitosan and cellulose are natural polymers with excellent biocompatibility, antimicrobial properties, and mechanical strength. When integrated with PBzs, these materials yield composites with improved functionality, making them suitable for diverse applications in biomedical, environmental, and industrial fields. Polyethylene glycol (PEG) is a polyether compound derived from petroleum with many applications, from industrial manufacturing to medicine. The integration of PEG into the PBz structure allows for improved processability, reduced brittleness, and unique functionalities such as water compatibility and biocompatibility.

### 3.1. Chitosan and Cellulose Grafted PBzs

**Figure 7:**
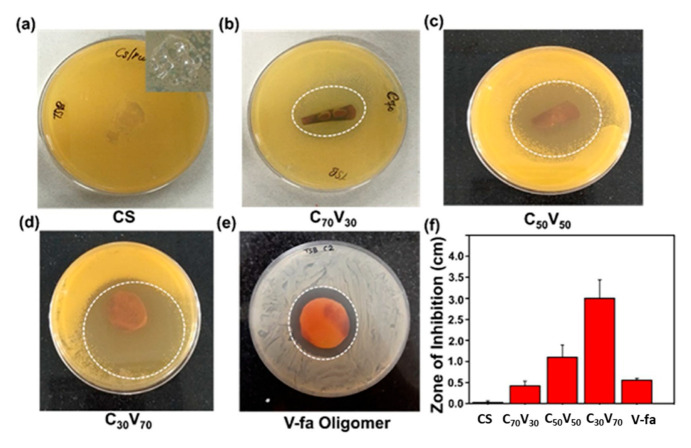
(**a**–**e**) Digital images of inhibition zone of CS and CS-graft-poly(V-fa) and V-fa oligomers on *S. aureus* (inset of CS after 24 h in media, without bacteria) and (**f**) diameter (cm) of the inhibition zone [38].

Chitosan-based PBz films represent an innovative hybrid material that integrates the advantageous properties of natural and synthetic polymers, offering enhanced antimicrobial activity and improved mechanical performance. Chitosan, derived from the deacetylation of chitin, is well known for its biocompatibility, biodegradability, and inherent antimicrobial properties. However, its practical applications are often constrained by its brittleness and limited mechanical and thermal stability [43,44,45,46,47]. To address these limitations, a fully biobased benzoxazine monomer, V-fa (synthesized from vanillin and furfurylamine) was grafted onto chitosan (CS) using benign “grafting to” Schiff base chemistry at various weight ratios (C_X_V_Y_) [38]. This approach led to reversible labile linkages, expansion of chitosan galleries, and the controlled leaching of phenolic species, which collectively enhanced the antibacterial activity against *S. aureus*. The antibacterial effect of the resulting films was approximately 125 times greater than that of bare CS films, V-fa alone, or oligomeric V-fa (Figure 7). Polybenzoxazine films are inherently hydrophobic, as confirmed by contact angle measurements. Similarly, the grafted V-fa structures on CS contributed to self-induced mechanical scrolling, with the C_30_V_70_ composition achieving a self-supported film in aqueous media. This was attributed to the higher V-fa content, which improved stability and mechanical integrity by increasing the hydrophobic domains in the hybrid framework. The C_30_V_70_ formulation exhibited the best antibacterial performance, inhibiting microbial growth over a large area, reflecting the enhanced mechanical and antimicrobial properties imparted by the grafted V-fa. Quantitative analysis and imaging of microbial cells demonstrated that nearly 95% of cells were nonviable in the C_70_V_30_ samples, providing direct evidence of the potent antimicrobial activity of CS-graft-poly(V-fa) films against *S. aureus*. These findings suggest that while V-fa alone is not as effective, its grafting onto CS and the formation of in situ ring-opened V-fa structures dramatically improve antibacterial performance, offering significant potential for advanced antimicrobial materials.

**Figure 8:**
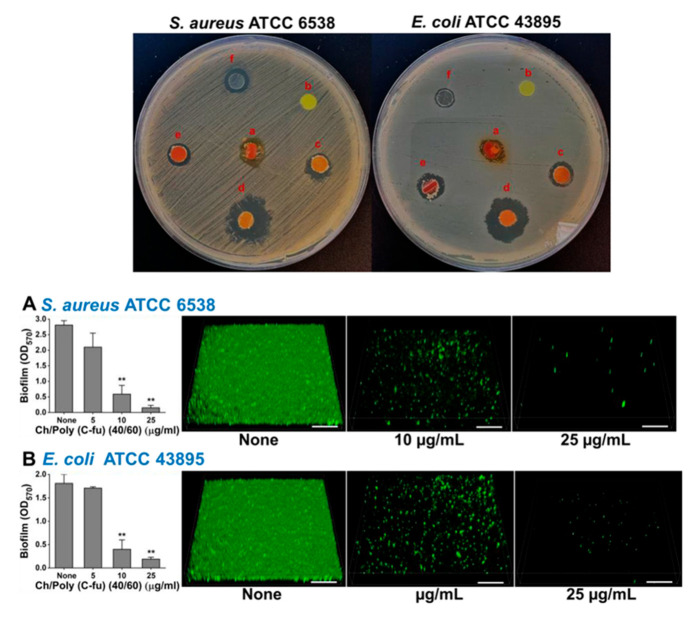
Antimicrobial activities of Ch/Poly (C-fu) blends with different C-fu contents: (**a**) 100%, (**b**) 80%, (**c**) 60%, (**d**) 40%, (**e**) 20%, and (**f**) 0%. (**A**,**B**) Biofilm formation observed by confocal laser microscopy for Ch/Poly (C-fu) (40/60) after 24 h in 96-well plates (scale bars represent 100 m) [36].

In a related study, chitosan (Ch) was blended with C-fu, a curcumin and furfurylamine-based benzoxazine monomer, to create hybrid materials [36]. The properties of the Ch/C-fu mixtures were evaluated after the ring-opening polymerization of the benzoxazine component. Antibacterial tests against both Gram-positive *S. aureus* ATCC 6538 and Gram-negative *E. coli* ATCC 43895 demonstrated that Ch/poly(C-fu) films were highly effective against these bacterial strains. Disk diffusion assays revealed that Ch/poly(C-fu) (40/60) films produced clear inhibition zones against both bacteria, measuring 18 ± 0.5 mm for *E. coli* and 18 ± 0.9 mm for *S. aureus* (Figure 8). The minimum inhibitory concentration (MIC) of Ch/poly(C-fu) (40/60) was determined to be 50 µg/mL for both bacterial strains. Moreover, the bactericidal efficacy increased as the benzoxazine content (C-fu) in the blend rose to 60% by weight, indicating a synergistic effect between polybenzoxazine (PBz) and chitosan at this specific ratio. Above or below this optimal concentration, the antibacterial activity either diminished or was not observed. In addition, Ch/poly(C-fu) (40/60) films at a concentration of 25 µg/mL effectively reduced the biomass and mean thickness of *S. aureus* and *E. coli* biofilms by over 95% and 97%, respectively, compared to untreated controls. These findings highlight the enhanced antibacterial properties and biofilm-reduction capabilities of Ch/poly(C-fu) films, making them promising candidates for antimicrobial and biofilm-preventive applications.

**Scheme 5:**
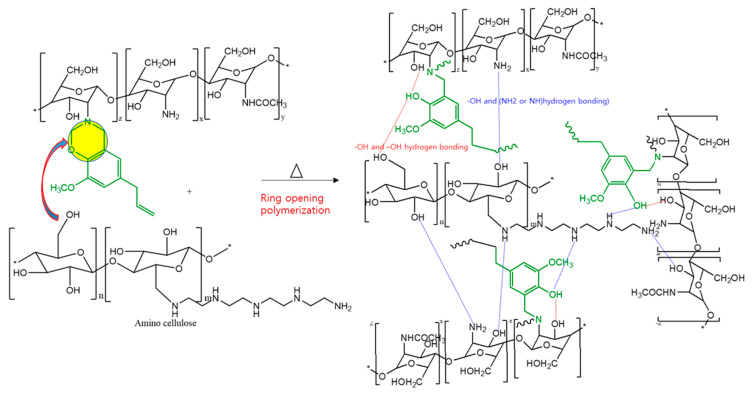
Mechanism of ring-opening polymerization of benzoxazine in E-ch/AC blends [40].

**Figure 9:**
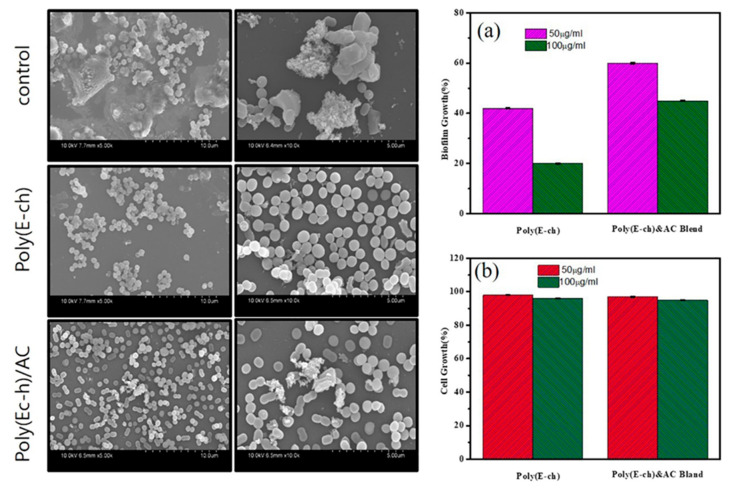
SEM images showing the effect of [poly(E-ch) and poly(E-ch)/AC] biofilms on *S. aureus*, [scale bar 10 μm (**left**) and 5 μm (**right**)]; (**a**) biofilm growth and (**b**) cell growth of [poly(E-ch) and poly(E-ch)/AC] with 50 and 100 μg/mL concentrations [41].

Cellulose, a glucose-based biopolymer abundant in plant cell walls, is widely distributed and features a high number of reactive hydroxyl groups. These hydroxyl groups enable extensive chemical modification, resulting in a variety of cellulose derivatives with tailored functional properties. Cellulose and its derivatives are known for forming transparent films with significant mechanical strength and excellent water absorption capabilities. Amino cellulose (AC), a derivative of cellulose with terminal –NH_2_ groups, is typically synthesized through the reaction of amine compounds with cellulose. AC exhibits excellent solubility and film-forming properties due to the abundant hydrophilic –OH and –NH_2_ groups, which enhance its solubility and make it an effective cross-linker for film production [48,49,50,51,52]. A novel class of bio-based polymer blends has been developed by combining a modified chitosan-based benzoxazine precursor (E-ch) with amino cellulose (AC) [41] (Scheme 5). AC contributes exceptional film-forming ability, biocompatibility, and biodegradability to the blend. The interaction between poly(E-ch) and AC involves strong hydrogen bonding, which significantly enhances the mechanical strength, thermal stability, and char yield of the resulting polymer films. Poly(E-ch)/AC (60/40) biofilm was tested for antimicrobial activity against *S. aureus* and antifungal efficacy against *C. albicans*. These biofilms effectively inhibited biofilm-related infections caused by both microorganisms. Interestingly, increased bio-activity was observed at lower concentrations (50 µg/mL), whereas reduced effects were noted at higher concentrations (100 µg/mL). Despite these concentration-dependent variations in antimicrobial activity, cell growth remained unaffected at both concentrations (Figure 9). These findings indicate that poly(E-ch)/AC (60/40) biofilms possess potent antimicrobial and antifungal properties, making them promising candidates for applications in infection prevention and management.

### 3.2. PEG and Copolymers of PEG

**Figure 10:**
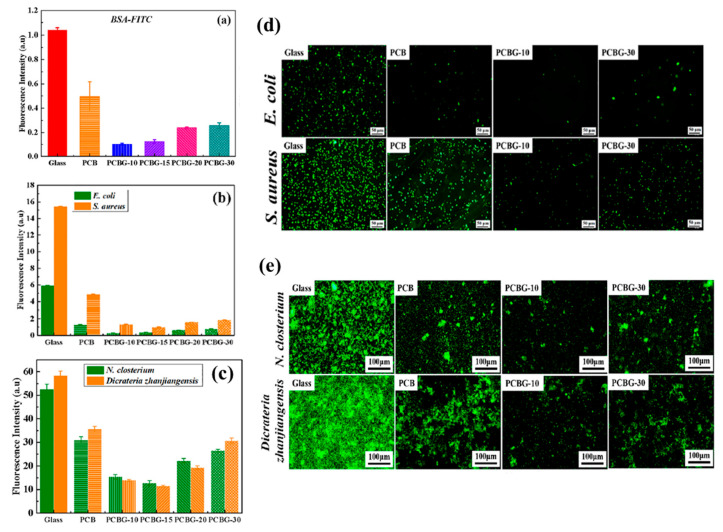
Fluorescence intensity of different surfaces after (**a**) protein adsorption test, (**b**) bacteria *E. coli* and *S. aureus* settled on different surfaces, (**c**) microalgae *N. closterium* and *Dicrateria zhanjiangensis* settled on different surfaces. (**d**) Fluorescence microscopic images of bacteria *E. coli* and *S. aureus* settled on different surfaces after 6 h of immersion in bacterial suspensions (scale bar 50 µm). (**e**) Fluorescence microscopic images of microalgae *N. closterium* and *Dicrateria zhanjiangensis* settled on different surfaces after 7 days of immersion in microalgae medium (scale bar 100 µm) [37].

PEG is an effective fouling-resistant polymer that enhances surface resistance to microbial attachment and settlement. PEG functions as both a functional polymer and a modiqfication agent, improving anti-biofouling properties through mechanisms such as hydration layer formation and steric repulsion. By forming a strong hydration layer between water molecules and PEG chains, or by the steric repulsion of the polymer chains, PEG-based materials effectively resist fouling from proteins, bacteria, cells, and other organisms [53,54,55,56]. In a recent study [37], hydrophilic PEG molecules were incorporated into a curcumin-based benzoxazine monomer (CB) to leverage the inherent properties of the resin while enhancing its anti-biofouling performance. The results demonstrated that the fouling repellency of the coatings was significantly influenced by the PEG content. The strong hydrated layer and interaction energy imparted by PEG contributed to superior fouling resistance. Among the various PEG loadings, PCBG-10 and PCBG-15 showed the highest efficiency in preventing bacterial attachment. For *E. coli* and *S. aureus*, PCBG-10 achieved anti-attachment efficiencies of 95.09% ± 0.14 and 91.25% ± 0.06, respectively, while PCBG-15 exhibited efficiencies of 93.06% ± 0.04 and 93.65% ± 0.20, respectively. The coatings were also tested against microalgae species *N. closterium* and *Dicrateria zhanjiangensis*, where PCBG-10 achieved antifouling efficiencies of 70.59% ± 0.02 and 76.09% ± 0.01, respectively, and PCBG-15 exhibited efficiencies of 75.59% ± 0.03 and 80.27% ± 0.01 (Figure 10). To further enhance the functional performance, a double-layer coating system was designed for combined anticorrosion and antifouling applications. The sub-layer consisted of PCB (curcumin-based PBzs), known for its excellent anticorrosion properties, while the up-layer comprised PCBG-10 or PCBG-15 (a hybrid polymer of PBzs and PEG with superior antifouling properties). The coatings, designated as P10 (PCB/PCBG-10) and P15 (PCB/PCBG-15), demonstrated exceptional long-term performance, maintaining strong anticorrosion barriers even after 180 days of immersion in various corrosive environments. These results suggest the efficacy of the dual-layer coating system in providing robust protection against both corrosion and biofouling.

**Figure 11:**
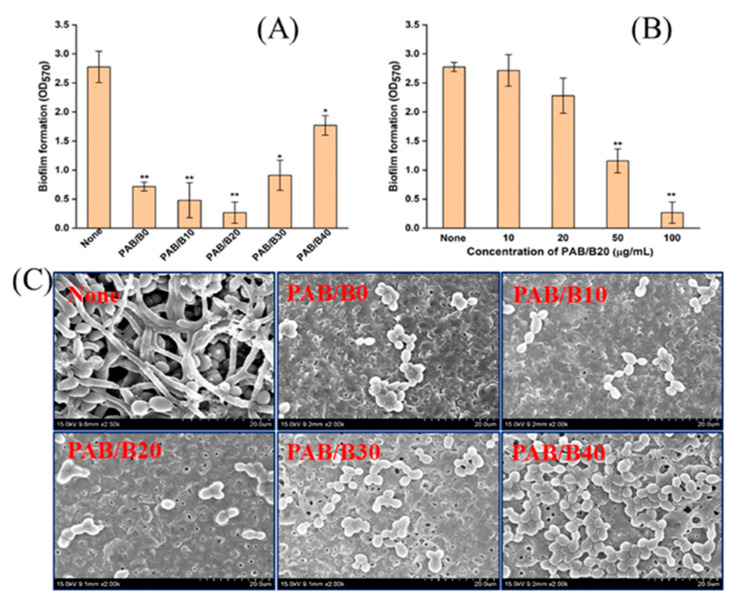
(**A**,**B**) Biofilm formation and (**C**) SEM images of LCS, PAB/B0, PAB/B10, PAB/B20, PAB/B30 and PAB/B40 [39].

In a related study [39], a series of polybenzoxazine/copolymer coatings (PAB/BX) incorporating arbutin and silane functionalization were developed, exhibiting dual functionalities: anti-corrosion and anti-biofouling properties. The synergistic combination of arbutin-based polybenzoxazine (PAB) and the copolymer (PEG-PPG-PEG) resulted in coatings with low water absorption and enhanced performance compared to their individual components. The PAB/BX polymers (PAB/B0, PAB/B10, PAB/B20, PAB/B30, and PAB/B40) were initially tested at a concentration of 100 μg/mL to evaluate their antimicrobial effects. Among these, PAB/B0, PAB/B10, and PAB/B20 showed significant reductions in biofilm mass, as measured by the crystal violet assay, with percentage reductions of 72.7%, 83.6%, and 90.9%, respectively. Notably, PAB/B20 demonstrated a dose-dependent reduction in biofilm formation, making it the most effective anti-biofilm agent among the tested polymers (Figure 11). Corrosion and biofilm studies revealed differing performance levels for anti-corrosion and anti-biofouling properties among the coatings. PAB/B10 exhibited the highest anti-corrosion performance, while PAB/B20 showed superior anti-biofouling properties. These differences likely arise from the distinct environmental conditions under which these properties are evaluated, highlighting the coatings’ variable behavior across applications. Both PAB/B10 and PAB/B20 offer valuable functionality but excel in different aspects, making them suitable for specific environments and requirements.

## 4. Antimicrobial Properties of Nanoengineered PBzs

Polybenzoxazine composites have gained considerable attention as versatile and high-performance materials, offering unique mechanical, thermal, and chemical properties suitable for a wide range of applications. Recent advancements in polybenzoxazine-based systems demonstrate their potential in two critical fields: dental restoration materials and marine antifouling coatings. Zirconia-reinforced polybenzoxazine composites address challenges like polymerization shrinkage, mechanical durability, and biocompatibility in dental applications. Simultaneously, urushiol-based polybenzoxazine (UOB) composites with in situ reduced silver nanoparticles (AgNPs) provide an eco-friendly alternative to toxic antifouling agents for marine industries. Together, these studies highlight the adaptability and transformative potential of polybenzoxazine composites in addressing both biomedical and environmental challenges.

### 4.1. Zirconia-Reinforced Polybenzoxazine

Zirconia-reinforced polybenzoxazine composites aim to overcome the issues associated with traditional materials, such as high polymerization shrinkage, limited durability, micro-crack formation, and restoration failure, by integrating the advantages of polybenzoxazine resins with the superior mechanical properties of zirconia particles. Lofti et al. [57] synthesized PBz composites reinforced with zirconia particles using a solvent-less approach, ensuring eco-friendly processing. The results showed that zirconia-reinforced PBz composites exhibited polymerization shrinkage as low as 2.8%, significantly lower than traditional dental polymers [3.3% for BisGMA (bisphenol A-glycidyl methacyrlate) and 8.9% for TEGDMA (triethylene glycol dimethyacrylate)]. Biocompatibility is critical for any dental material. Zirconia-reinforced polybenzoxazine composites demonstrated excellent biocompatibility in cellular studies. MTT assays showed over 97% cell viability, and SEM images confirmed robust cell adhesion and proliferation on the composite surface. These results suggest that the material not only supports tissue integration but also provides a favorable environment for long-term dental restoration.

### 4.2. Urushiol-Based Polybenzoxazine with Silver Nanoparticle

**Figure 12:**
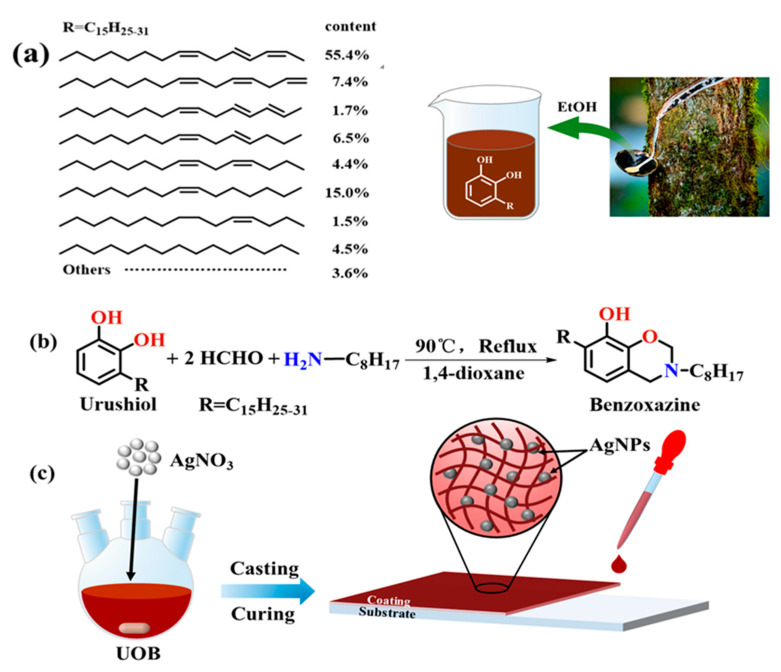
(**a**) Collection of raw lacquer and chemical structure of urushiol, (**b**) synthetic route of urushiol-based benzoxazine, and (**c**) preparation of UOB/AgNPs composites and UOHP/AgNPs composite coatings [58].

Marine biofouling, caused by the accumulation of microorganisms, algae, and barnacles on submerged surfaces, imposes significant economic and environmental burdens on maritime industries. Traditional antifouling solutions, such as tributyltin- and copper-based coatings, effectively reduce biofouling but release toxic substances that harm marine ecosystems. To address this, urushiol-based polybenzoxazine (UOB) composites with silver nanoparticles (AgNPs) [58] have been developed as a sustainable and efficient antifouling solution (Figure 12).

**Figure 13:**
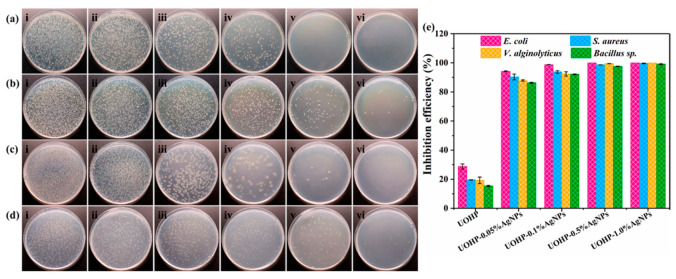
Digital photographs of antibacterial test towards typical (**a**) Gram-negative bacteria *E. coli*, (**b**) Gram-positive bacteria *S. aureus*, marine bacterial (**c**) *V. alginolyticus*, and (**d**) *Bacillus* sp. after 24 h of incubation on (**i**) BG, (**ii**) UOHP, (**iii**) UOHP-0.05%AgNPs, (**iv**) UOHP-0.1%AgNPs, (**v**) UOHP-0.5%AgNPs, and (**vi**) UOHP-1.0%AgNPs. (**e**) The inhibition efficiency of UOHP and UOHP/AgNP composite coatings relative to BG towards *E. coli* and *S. aureus*, *V. alginolyticus*, and *Bacillus* sp. [58].

Urushiol, a natural product derived from lacquer trees, was used as both a polymer matrix and a reducing agent to synthesize AgNPs via in situ reduction. This method avoids the need for external reducing agents, simplifying the process and reducing environmental risks. The phenolic hydroxyl groups in urushiol generate semiquinone radicals upon oxidation, which reduce silver ions to AgNPs. UOB serves as a reducing agent for synthesizing silver nanoparticles (AgNPs), a dispersant to prevent AgNP agglomeration, and a film-forming substrate that creates a low-surface-energy coating with fouling release properties. Meanwhile, AgNPs contribute as an antifouling agent to repel and eliminate fouling organisms. The synergistic combination of organic polymers and inorganic nanoparticles in these coatings enhances antifouling performance through improved nanoparticle dispersion and simple fabrication processes. The UOB/AgNP composite coatings demonstrated exceptional antimicrobial performance. Tests against Gram-positive (*Staphylococcus aureus*) and Gram-negative (*Escherichia coli*) bacteria, as well as marine bacteria such as *Vibrio alginolyticus*, showed nearly 100% inhibition at AgNP concentrations as low as 1% (Figure 13).

**Scheme 6:**
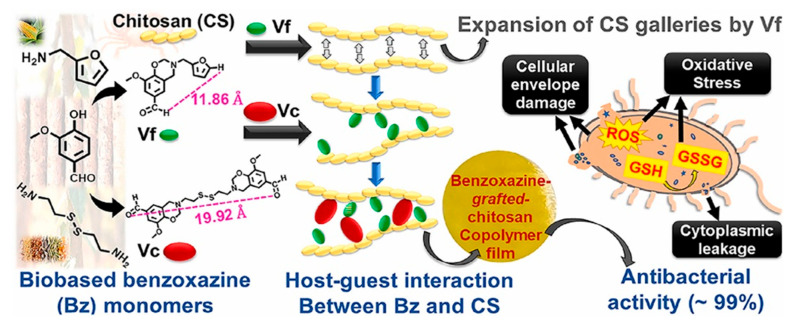
Schematic image and mechanism of antimicrobial action and cell interruption of polybenzoxazines [59].

The antimicrobial mechanism of AgNPs primarily involves the penetration of AgNPs and Ag^+^ ions through bacterial cell membranes, disrupting bacterial metabolism and causing oxidative stress. The UOHP coating, with its low surface energy, resists bacterial adhesion, allowing bacteria to be easily washed away by PBS. The UOHP/AgNP composite coatings further enhance this effect by incorporating AgNPs, which effectively inhibit bacterial proliferation and reduce their numbers. This synergistic combination of AgNPs’ antimicrobial properties and the low surface energy of UOHP minimizes bacterial adhesion, with bacterial presence decreasing as the concentration of AgNPs increases. The antimicrobial effects were particularly pronounced against Gram-negative bacteria, which have thinner cell walls compared to Gram-positive strains, allowing easier penetration of AgNPs. In addition to antimicrobial efficacy, the UOB/AgNP coatings exhibited significant antifouling properties. Tests against marine microalgae such as *Nitzschia closterium* and *Dicrateria zhanjiangensis* showed marked reductions in algal adhesion compared to control surfaces. The combination of AgNPs’ antimicrobial activity and the low surface energy of the UOB matrix created a synergistic effect that effectively deterred biofouling. Notably, the coatings required only minimal AgNP concentrations (≤1 wt%) to achieve these results, reducing potential environmental impact. The eco-friendliness of UOB/AgNP coatings is a major advantage. Unlike conventional antifouling systems that release toxic substances, these coatings leverage sustainable materials and processes. The in-situ reduction method ensures efficient AgNP incorporation without harmful byproducts, while the low nanoparticle content minimizes ecological risks associated with leaching. This makes UOB/AgNP coatings a viable alternative for marine vessels, aquaculture equipment, and offshore structures (Scheme 6).

## 5. Applications of PBzs with Antimicrobial Properties

PBzs with excellent antimicrobial properties find applications in several fields including coatings and paints, medical devices, food packaging, water treatment, and the textile industry. PBzs serve as protective coatings in marine and industrial settings, leveraging their antimicrobial and fouling-resistant properties to inhibit biofilm formation and microbial corrosion, thereby enhancing the durability and lifespan of the coated surfaces. They also play a crucial role in the healthcare industry by serving as antimicrobial coatings for various medical devices, including catheters, implants, and surgical instruments. These coatings address the critical issue of hospital-acquired infections (HAIs), which are often caused by bacterial colonization on medical device surfaces. The antimicrobial properties of PBzs are utilized in active food packaging to enhance shelf life and ensure food safety by effectively reducing microbial contamination. PBz-based membranes and coatings are employed in water filtration systems to inhibit bacterial and fungal growth, thereby enhancing water quality and extending the durability of filtration equipment. Antimicrobial PBzs are incorporated into fabrics to create odor-resistant and hygienic materials, making them ideal for applications in sportswear and medical textiles.

## 6. Drawbacks and Limitations

Despite their extensive applications, the use of PBzs is accompanied by several drawbacks, limitations, and critical considerations. Their high curing temperatures (180–250 °C) can restrict their use in temperature-sensitive environments. Incorporating antimicrobial agents, such as nanoparticles or organic molecules, often requires specialized synthesis or surface modification techniques to ensure uniform distribution and compatibility with the PBz matrix. The type, concentration, and dispersion of antimicrobial agents are crucial for effective microbial inhibition, and testing under various environmental conditions is necessary to confirm broad-spectrum activity. The production of PBzs and their composites can be costly, especially when advanced antimicrobial agents like silver nanoparticles or functionalized compounds are involved. Certain additives, such as metal nanoparticles, may pose environmental and health risks. Using eco-friendly antimicrobial agents is essential to mitigate these concerns. Additionally, the non-biodegradable nature of PBzs presents challenges for disposal and recycling, raising sustainability issues. Over time, antimicrobial properties may decline due to leaching or degradation of the active agents, which necessitates careful design to retain efficacy throughout the polymer’s lifespan. Customization of PBz formulations is often required to address specific application needs, such as in medical devices, coatings, or packaging materials.

## 7. Conclusions and Future Perspectives

PBzs have emerged as a transformative class of thermosetting polymers, with properties that align seamlessly with the needs of high-performance applications in diverse sectors such as aerospace, electronics, coatings, adhesives, and biomedical fields. Their exceptional thermal stability, mechanical strength, flame retardancy, and chemical resistance underscore their versatility and relevance in both industrial and scientific domains. Beyond these traditional attributes, the antimicrobial potential of PBzs represents a promising frontier, driven by innovative synthesis strategies and the integration of bio-active compounds. The antimicrobial efficacy of PBzs is rooted in their unique structural characteristics, including hydrophobic surfaces and functional group modifications that disrupt microbial processes. By tailoring PBzs with components such as amines, phenolic moieties, quaternary ammonium salts, or metal nanoparticles, researchers have significantly enhanced their ability to combat bacteria, fungi, and viruses. This capability is particularly relevant for applications requiring sterility, such as medical devices, coatings, and water purification systems. The advent of bio-based PBzs marks a pivotal step toward sustainable material development. Utilizing renewable resources like curcumin, vanillin, eugenol, and other naturally derived compounds, bio-based PBzs not only exhibit remarkable antimicrobial properties but also address environmental concerns associated with petroleum-based polymers. The bio-based benzoxazines are categorized into partially and fully bio-based types depending on the extent of renewable content used in their synthesis. For enhanced antimicrobial properties, fully bio-based Bzo will be more effective than partially bio-based Bzo. Other properties of bio-based Bzo include lowering curing temperature, film-forming ability, and solubility of the final product in an eco-friendly solvent. When PBzs are designed with these factors will impart antimicrobial property and environmental friendliness. Blending with biopolymers enhances the antimicrobial properties of PBzs to the next level. Almost all reports show that PBzs with adjustable ratios of biopolymers will be more effective than the neat polymer (PBz). These types of PBzs find applications mainly in the coating field, either marine or medical. When expecting antimicrobial properties with improved mechanical stability or hardness, PBz nanocomposites with biocompatible nanoparticles such as silver, zirconia, zinc, and copper are used. Depending upon the final application, by utilizing benzoxazine chemistry and incorporating suitable bio-materials, the applications of bio-based PBzs are widened. In conclusion, the diverse capabilities of PBzs, coupled with their adaptability through molecular design and hybridization, position them as a cornerstone of material innovation. Their ability to offer tailored solutions for antimicrobial applications while meeting stringent performance criteria ensures their continued relevance in advancing global material science. Future research should focus on scaling sustainable production methods, enhancing the environmental compatibility of PBzs, and expanding their utility across emerging fields like nanomedicine, smart materials, and sustainable packaging. With continued innovation, PBzs are poised to play a transformative role in addressing both technical challenges and environmental concerns in the years ahead.
